# Hypoxia-Inducible Factor 2-Alpha Mediated Gene Sets Differentiate Pulmonary Arterial Hypertension

**DOI:** 10.3389/fcell.2021.701247

**Published:** 2021-08-05

**Authors:** Jinsheng Zhu, Li Zhao, Yadan Hu, Guoqi Cui, Ang Luo, Changlei Bao, Ying Han, Tong Zhou, Wenju Lu, Jian Wang, Stephen M. Black, Haiyang Tang

**Affiliations:** ^1^College of Veterinary Medicine, Northwest A&F University, Xianyang, China; ^2^Department of Physiology, Nanjing Medical University, Nanjing, China; ^3^Department of Physiology and Cell Biology, University of Nevada School of Medicine, Reno, NV, United States; ^4^State Key Laboratory of Respiratory Disease, Guangdong Key Laboratory of Vascular Disease, National Clinical Research Center for Respiratory Disease, Guangzhou Institute of Respiratory Health, The First Affiliated Hospital of Guangzhou Medical University, Guangzhou, China; ^5^Department of Cellular Biology and Pharmacology, Herbert Wertheim College of Medicine, Miami, FL, United States; ^6^Department of Environmental Health Sciences, Robert Stempel College of Public Health and Social Work, Miami, FL, United States; ^7^Center for Translational Science, Florida International University, Port St. Lucie, FL, United States

**Keywords:** HIF2α, PHD2, pulmonary arterial hypertension, hypoxia, microarray

## Abstract

**Objectives:**

HIF2α is of vital importance in the regulation of endothelial dysfunction, cell proliferation, migration, and pulmonary vascular remodeling in pulmonary hypertension. Our previous studies demonstrated that conditional and inducible deletion of HIF2α in mouse lung endothelial cells, dramatically protected the mice against vascular remodeling and the development of pulmonary arterial hypertension (PAH). Here, we provide a novel transcriptome insight into the impact of HIF2α in PAH pathogenesis and the potential to use HIF2α-mediated gene sets to differentiate PAH human subjects.

**Methods:**

Using transcriptome data, we first tapped the value of the difference in gene expression profile between wild type (WT) and *Hif2a* knockdown (KD) cell lines. We considered the deregulated genes between WT and *Hif2a*-KD cells as HIF2α influenced genes. By examining the lung tissue transcriptome data set with nine controls and eight PAH patients, we evaluated the HIF2α regulatory network in PAH pathogenesis to further determine the identification ability of HIF2α-mediated gene sets in human PAH subjects. On the other hand, using peripheral blood mononuclear cells (PBMCs) transcriptome data from PAH patients and healthy controls, we further validated the potential of the HIF2α-mediated PBMC gene sets as a possible diagnostic tool for PAH. To verify the ability of HIF2α-mediated gene sets for the identification of PAH, endothelial cell-specific *Phd2* knockout mice with spontaneous pulmonary hypertension were used for reverse validation experiments.

**Results:**

19 identified GO biological process terms were significantly correlated with the genes down-regulated in *Hif2a*-KD cells, all of which are strongly related to the PAH pathogenesis. We further assessed the discriminative power of these HIF2α-mediated gene sets in PAH human subjects. We found that the expression profile of the HIF2α-mediated gene sets in lung tissues and PBMCs were differentiated both between controls and PAH patients. Further, a significant positive correlation was observed between hypoxia and *Phd2* deficiency mediated gene set expression profiles. As expected, 7 of the 19 significantly down-regulated GO terms in *Hif2a*-KD cells were found to overlap with the up-regulated GO gene sets in *Phd2*^*EC*–/–^ mice compared to WT controls, suggesting opposing effects of HIF2α and PHD2 on PAH pathogenesis.

**Conclusion:**

HIF2α*-*mediated gene sets may be used to differentiate pulmonary arterial hypertension.

## Introduction

Pulmonary artery hypertension (PAH) is a progressive and lethal disease caused by multiple pulmonary vascular disorders. Patients with PAH eventually died from right heart failure. Endothelial dysfunction, sustained vasoconstriction and vascular remodeling of pulmonary arterioles are the major characteristics of PAH, all of which act to narrow the blood vessels and increase pulmonary vascular resistance (PVR) and pulmonary artery pressure (PAP). Most patients with PAH have an insidious onset initially with no specific symptoms, however, with the further increase of PAP, asthma, chest pain, dizziness occur. Severe cases of right heart failure include edema of the lower extremities, enlargement of the liver, and even ascites or pleural effusion. Once the symptoms of right heart failure appear, the prognosis of patients is poor. At present, the drugs targeting PAH in clinical usage are suboptimal. In general, the median survival rate after diagnosis of idiopathic pulmonary hypertension is only 2–3 years. Most importantly, PH cannot be measured by conventional sphygmomanometers and most PH is found by echocardiography after patients are already exhibiting severe symptoms, which delays treatment. Therefore, it is critical that earlier methods of PAH diagnosis are developed.

In pathological conditions such as PAH, one of the common phenomenon is oxygen utilization rate decrease. Pulmonary hypertension caused by lung disease and/or hypoxia (WHO group 3 PH) has a large population, which cannot be ignored. A large amount of evidence supports the crucial role of hypoxia inducible factors (HIFs) in chronic hypoxia induced PH (Brusselmans et al., 2003; Kim et al., 2013; Cowburn et al., 2016; Dai et al., 2016). HIFs are a class of transcription factors sensitive to oxygen concentration, which are mainly responsible for regulating the adaptability of the body to hypoxia (Prabhakar and Semenza, 2012). HIFs, including HIF-1 and HIF-2 are involved in the regulation of cell proliferation, migration, and pulmonary vascular remodeling in PH (Voelkel and Tuder, 2000; Brusselmans et al., 2003; Prabhakar and Semenza, 2012). More importantly, HIF1α and HIF2α, particularly HIF2α in pulmonary artery endothelial cells, plays a major role in the development of PAH (Skuli et al., 2009; Kim et al., 2013; Ball et al., 2014; Dai et al., 2016; Kapitsinou et al., 2016). HIFs are regulated by HIF-prolyl hydroxylases (PHDs) through the hydroxylation of conserved proline residues. Hydroxylated HIFs are then degraded by the proteasome via the von Hipel-Lindau ubiquitination complex. Under hypoxia, the hydroxylation reaction is attenuated allowing HIFs to escape degradation, which leads to their nuclear translocation, heterodimerization with HIF1β, and increased expression of hypoxia-induced genes (Bishop and Ratcliffe, 2015). PHD2 is the most important isoenzyme under normoxic conditions, and is involved in various hypoxia-affected processes, such as angiogenesis and cardiac function. We, and others, have already reported that the *Phd2*^*EC*–/–^ mice, the endothelial conditional knockout mice, developed spontaneous PH with severe pulmonary vascular remodeling and occlusive pulmonary vascular lesions under normoxic conditions (Dai et al., 2016; Kapitsinou et al., 2016; Tang et al., 2018), while heterozygotes, *Phd2*^*EC*–/–^ mice developed mild PH symptoms. Significant progress has also been made on the mechanism of HIF2α in the pathogenesis of PAH and it appears that HIF2α causes PH by inducing endothelial-to-mesenchymal transition (Endo-MT), a phenomenon of endothelial cells transforming into fibroblast phenotype, which exacerbates pulmonary vascular lesions (Tang et al., 2018).

Preclinical studies have also begun to evaluate small molecule HIF2α inhibitors as potential candidates for PAH treatment. The transcriptional activity of HIF2α is activated by binding with ARNT while Gardner and his colleagues have found that the hydrophobic structure in the PAS-B domain of HIF2α that can be inactivated by specific small molecule entry, which leads to the dissociation of the HIF2α and ARNT heterodimer. Ultimately the DNA-binding capacity of HIF2A is abolished (Scheuermann et al., 2009). A series of HIF2α inhibitors for cancer therapy have been produced using this method, including PT2385, PT2399, and PT2576. The advantages of high specificity and oral high bioavailability make them of high clinical value. These HIF2α inhibitors show high safety and effectiveness in both animal experiments and clinical trials, which can significantly inhibit tumor growth in patients with metastatic clear cell renal cell carcinoma (ccRCC), with proven safety and high dose tolerance (Chen et al., 2016; Cho et al., 2016).

Since HIF2α is an important cause of pulmonary artery remodeling during PH, preclinical studies of HIF2α inhibitors for PH therapy have been evaluated. Published data shows that PT2567 significantly reduced hemodynamic parameters related to the development of PH in Sugen 5416/hypoxia- rats (Macias et al., 2020). These results suggest that HIF2α could be an early biomarker of PAH and that HIF2α inhibitors might provide a promising new approach for the treatment of PAH. There is evidence that HIF-2α and its target genes were upregulated in lung tissue and pulmonary artery endothelial cells (PAECs) from idiopathic PAH patients and a variety of rodent PH models (Dai et al., 2018; Tang et al., 2018). These findings suggest that HIF2α plays an important role not only in hypoxia-induced PH, but also in other types of PH. However, due to the heterogeneity of PH it is vital to specifically utilize the mots efficacious therapeutic agents. Therefore, in this study we aim to investigate the gene expression of HIF2α in PAH in order to determine if it was possible to identify a useful diagnostic method to differentiate PAH human subjects.

Because highly reproducible gene expression pattern can be acquired from the mechanism-associated gene sets related to PH development from transcriptional analysis (Abraham et al., 2010; Yang et al., 2012; Gardeux et al., 2014), We focused on the global gene expression pattern associated with the HIF2α rather than examining the expression abundance of individual genes. We compared the Gene Ontology (Bertout et al., 2009) gene set (Ashburner et al., 2000) expression pattern between wild type (WT) and *Hif2a* knockdown (KD) von Hippel-Lindau (VHL) deficient cells. The differentially expressed GO gene sets were deemed as HIF2α-mediated. We further validated the potential of the HIF2α-mediated gene sets as a possible diagnostic tool for PAH by analyzing the transcriptome data in lungs and PBMCs of PAH patients and healthy controls. To verify the ability of HIF2α-mediated gene sets for the identification of PAH, endothelial cell-specific *Phd2* KO (*Phd2*^*EC*–/–^) mice were generated, and their lung tissues were analyzed by transcriptome sequencing. Comparing the differences and similarities of gene expression and biological processes among WT, hypoxia and *Phd2*^*EC*–/–^ mice lung tissues we found that differentially expressed GO genes associated with PH were mediated by HIF2α. Together, these HIF2α-mediated gene sets were effectively able to differentiate PAH patients from controls.

## Materials and Methods

### Generation of Phd2 Gene Endothelial Cell-Specific Knockout Mice

The endothelial cell specific-knockout (Gong et al., 2015) mouse lines were generated by Cre-LOX technique according to the methods previously described (Bishop and Ratcliffe, 2015). The *Phd2* floxed mice were hybridized with *Tie2-Cre* mice to produce *Tie2-Cre^+^/Phd2^*flox/flox*^* mice, which were named endothelial cell-specific *Phd2* knockout mice (*Phd2*^*EC*–/–^). The expression of Cre recombinase was regulated by the *Tie2* promoter.

### Western Blotting

Western blotting was done as previously described (Tang et al., 2018). Antibodies that have been used are anti-HIF2α (Cat No. ab199, Abcam), anti-PHD2 (Cat No. 66589-1-Ig, Proteintech), and anti-β-actin (Cat No. 4970, Cell Signaling).

### Hemodynamic Measurements

All animal studies were approved by the Institutional Animal Care and Use Committee (IACUC) of Guangzhou Medical University and the procedures applied in the studies were carried out in accordance with the National Institutes of Health guidelines for use of live animals. Right ventricular pressure (RVP) was collected by MPVS ULTRA^TM^ system in real time and right ventricular systolic pressure (RVSP) was calculated in LabChart8 application. The Fulton Index was measured as a key indicator of RV hypertrophy (RVH). Paraffin embedded sections of mice lung tissues were used for morphological analysis. The sections were stained with hematoxylin and eosin (*H&E*) to evaluate the degree of vascular remodeling by calculating the thickness of pulmonary artery walls.

### Transcriptome Data Processing

The whole-genome gene expression data packages of WT and *Hif2a*-KD VHL-deficient human cell renal cell carcinoma (ccRCC) cell line A498 were acquired from the Gene Expression Omnibus (GEO) database (Edgar et al., 2002) (GEO accession: GSE16622; Bertout et al., 2009). Affymetrix Mouse Gene 2.0 ST Array (Affymetrix, Inc., Santa Clara, CA) has been used to profile mRNA expression abundance in *Phd2*^*EC*–/–^ mice lung tissues. Briefly, the Affymetrix arrays were analyzed using the Affymetrix Power Tools. A robust multi-array averaging method was used to summarize the probe set expression signals (Gong et al., 2015) algorithm and log_2_-transformed with a median polish. The transcript was deemed reliably expressed if the Affymetrix implemented detection above ground (DABG) *P*-value was < 0.01 in all samples from at least one group. We focused on genes with unique annotations. Genes on X and Y chromosomes were discarded to avoid potential confounding factors caused by sex. Significance analysis of microarrays (SAM) algorithm, implemented in the samr library of the R Statistical Package, was applied to identify the differentially expressed genes between WT and *Hif2a*-KD cells. Transcripts with a fold-change (FC) > 1.3 and false discovery rate (FDR) < 1% were deemed differentially expressed. Affymetrix Mouse Gene 2.0 ST Array has been used to analyze mRNA expression abundance in *Phd2*^*EC*–/–^ mice lung tissues.

To understand the role of HIF2α in PAH pathogenesis, four transcriptome data sets of human samples were acquired from the GEO database: GSE48149, GSE15197, and GSE53408 from lung tissue and GSE22356 from PBMCs. These specific data sets been selected were dependent on the availability of annotated patient classification. For all these data sets, we aggregated genes into gene set-level mechanisms using GO (Ashburner et al., 2000) annotations.

### Computing Gene Set Score Using Human Transcriptome Data

In order to calculate a gene set score for each GO biological process term, the Functional Analysis of Individual Microarray Expression (FAIME) algorithm was adopted (Yang et al., 2012). Gene set scores computed by the FAIME tool made the gene expression of individual samples rank-weighted, which transferred each sample’s transcriptome profile into pathway-level data (Yang et al., 2012). A higher gene set score indicates an overall up-regulation of a given GO term.

## Results

### Deregulated Gene Sets in Hif2a-KD Cells

**FIGURE 1:**
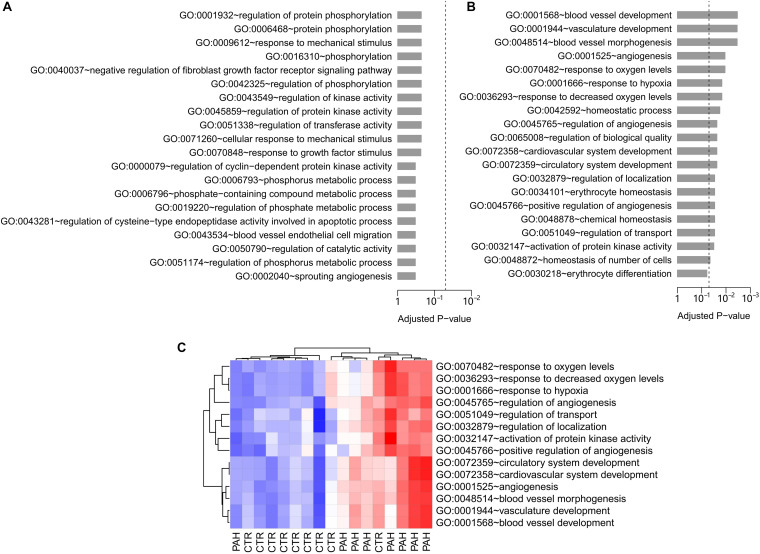
GO biological process analysis in *Hif2a*-KD VHL-deficient human ccRCC cells and PAH patients. **(A)** The top 20 GO biological process terms associated with the genes up-regulated in *Hif2a*-KD VHL-deficient human ccRCC cells. Fisher’s exact test was adopted to calculate the *P*-values which was then corrected by Benjamini-Hochberg procedure. The significance level of 0.05 was denoted by the dash line. **(B)** The top 20 GO biological process terms associated with the genes down-regulated in *Hif2a*-KD cells. The *P*-values were calculated by Fisher’s exact test and corrected by Benjamini-Hochberg procedure. The dash line denotes the significance level of 0.05. All the GO gene sets listed are statistically significant except the last term, “GO:0030218∼erythrocyte differentiation.” **(C)** Gene set score heatmap of the HIF2α-mediated GO biological process terms in human lungs. In total, 14 GO terms up-regulated in lung tissues from PAH patients were listed. Red and blue represent relative up-regulation and down-regulation of gene expression, respectively.

To infer the gene sets potentially mediated by HIF2α, we tapped the value of the difference in gene expression pattern between WT and *Hif2a-*KD cell lines. A microarray data set containing both WT and *Hif2a-*KD VHL-deficient human ccRCC cells gene expression abundance were obtained from the GEO database (GEO accession: GSE16622; Bertout et al., 2009). Within the specified significance level range (FDR < 1% and FC > 1.3), 193 genes were found to be up-regulated in *Hif2a-*KD cells while 353 genes were down-regulated. These differentially expressed genes were presented in Supplementary material (Supplementary Tables 1, 2). We hold the opinion that these deregulated genes were influenced by HIF2α. Meanwhile, the enriched GO biological processes terms (Ashburner et al., 2000) among the HIF2α influenced genes were analyzed. No significantly enriched GO terms were existed in the genes up-regulated in *Hif2a-*KD cells (Figure 1A). However, 19 GO biological process terms were identified as significantly associated with genes down-regulated in *Hif2a-*KD cells. These included “blood vessel development,” “angiogenesis,” “response to decreased oxygen level,” “erythrocyte homeostasis,” etc. (Figure 1B), all of which are strongly related to the pathogenesis of PAH.

### Expression Profile of HIF2α-Mediated Gene Sets in Lung Tissue Differentiates Between Control and Patients With PAH

To further determine the regulatory networks of HIF2α in PAH pathogenesis, we assessed the discriminative power of HIF2α-mediated gene sets in PAH human subjects. To accomplish this we examined a lung tissue transcriptome data set with nine controls and eight PAH patients (GEO accession: GSE48149) (Hsu et al., 2011). For each GO biological process terms, we computed a gene set score by using the FAIME algorithm (Yang et al., 2012). The higher the score of gene set, the higher the overall expression level of specific GO gene set. 14 of the 19 significantly down-regulated GO terms in *Hif2a*-KD cells were found to overlap with the up-regulated GO gene sets (*t*-test: *P* < 0.05) in PAH patients compared to controls, which was statistically significant (cumulative hypergeometric test: *P* = 9.4 × 10^–4^). The gene set score heat map of the overlapped GO terms generated by unsupervised hierarchical cluster analysis reveals a clear separation between controls and PAH patients (Figure 1C). Together our results suggest that HIF2α plays a regulatory role in PAH. Therefore, we deemed the 14 overlapped GO terms “HIF2α-mediated” in lung. Physiologic parameter and the differentially expressed genes in the citation (Hsu et al., 2011) between IPAH group and Control group were presented in Supplementary Material (Supplementary Tables 3–5 and Supplementary Figure 1).

**FIGURE 2:**
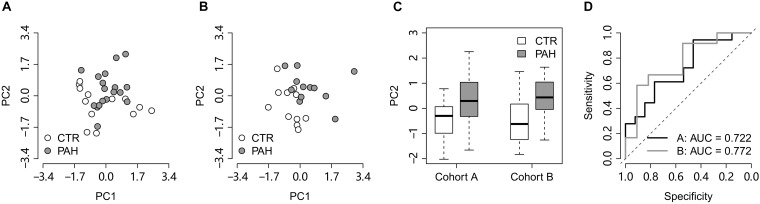
The HIF2α-mediated GO terms distinguish PAH patients from controls in the lung validation data sets. **(A,B)** Principal component analysis on the HIF2α-mediated GO terms in the validation cohorts **(A,B)**, respectively. PC1 and PC2 represent the first principal component and the second principal component, respectively. **(C)** PC2 differentiates the PAH patients from the controls in the validation cohorts. **(D)** The ROC curves of PC2 in distinguishing PAH patients from controls in the validation cohorts. CTR, control; PAH, pulmonary arterial hypertension.

We next tested the classification power of the 14 HIF2α-mediated GO terms in distinguishing PAH patients from controls in two validation lung transcriptome data sets: the cohort A (GEO accession: GSE15197; Rajkumar et al., 2010) containing 13 controls and 18 PAH patients and the cohort B (GEO accession: GSE53408; Zhao et al., 2014) with 11 controls and 12 PAH patients. Principal component analysis (PCA) indicates that the FAIME score of the HIF2α-mediated GO gene sets differentiate PAH patients from controls in both the validation cohorts (Figures 2A,B). There was a significant difference in the second principal component (PC2) between controls and PAH patients (*t*-test: *P* = 2.2 × 10^–2^ and *P* = 2.6 × 10^–2^ for the cohort A and B respectively) (Figure 2C). Area under the receiver operating characteristic curve (AUC) was adopted to evaluate the classification performance of PC2 which were 0.722 and 0.772 for the cohorts A and B, respectively (Figure 2D).

**FIGURE 3:**
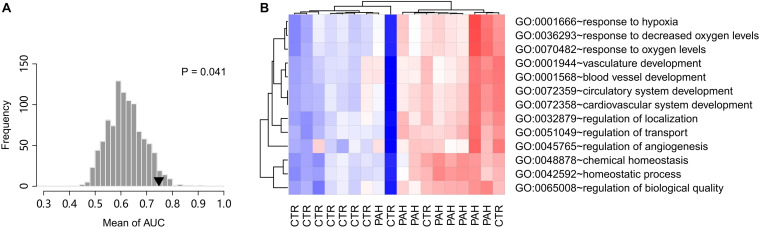
Gene set score heatmap of the HIF2α-mediated GO biological process terms in human PBMCs. **(A)** Superior predictive power of the HIF2α-mediated GO terms compared with randomized pattern. The histogram shows the distribution of the mean of AUC (both the validation cohorts **A,B**) for the 1,000 resampled GO term sets (The same size as the HIF2α-mediated GO terms, i.e., 14 GO terms). The mean of AUC of the 14 HIF2α-mediated GO terms is marked with black triangle. Right-tailed *P*-value was generated from sampling distribution. **(B)** In total, 13 GO terms up-regulated in PBMCs from PAH patients were listed. Red and blue represent relative up-regulation and down-regulation of gene expression, respectively.

A resampling test has been conducted to test whether the discriminative power of the 14 HIF2α-mediated GO terms was significantly better than random gene sets. We obtained 1,000 random set of GO terms by randomly selecting 14 GO biological process terms (the same size as the 14 HIF2α-mediated GO terms) from the GO database and calculated the AUC for each random GO term set. Our alternative hypothesis was that the AUCs of the 14 HIF2α-mediated GO terms ought to be more positive than the accidental expectation if the predictive power of the 14 HIF2α-mediated GO terms was significantly better than the random GO term sets. Our analysis shows that the predictive power of null hypothesis is accidental and should be rejected. The mean of AUC (for cohorts A and B) derived from the 14 HIF2α-mediated GO terms was significantly better than that of the random GO term sets (Right-tailed: *P* = 0.041) (Figure 3A), which suggests the non-random predictive power of the 14 HIF2α-mediated GO terms.

### PBMC Expression Profile of HIF2α-Mediated Gene Sets Differentiate PAH Patients From Controls

Chronic exposure of PBMCs to a PAH vascular environment may be reflected by transcriptome changes in these cells. Thus, we further tested the hypothesis that PBMC expression profile of HIF2α-mediated gene sets may differentiate between controls and PAH patients. Accordingly, we looked into a PBMC transcriptome data set with 10 controls and eight PAH patients (GEO accession: GSE22356; Risbano et al., 2010). Gene sets score was computed for each GO biological process terms based on the PBMC gene expression data, using the FAIME algorithm. 13 of the 19 significantly down-regulated GO terms in *Hif2a*-KD cells were found to overlap with the up-regulated GO gene sets (*t*-test: *P* < 0.05) in PAH patients vs. controls, which was statistically significant (cumulative hypergeometric test: *P* = 7.3 × 10^–7^). The PBMC based gene set score heat map of the 13 overlapped GO terms reveals a clear separation between controls and PAH patients (Figure 3B), which suggests that the differences exist in PBMC expression of the HIF2α-mediated gene sets can be used as a biomarker of PAH to identify high-risk population and promote early diagnosis.

### HIF2α-Mediated Molecular Signaling Events Involved in the Development of Phd2-KO Mediated PH

**FIGURE 4:**
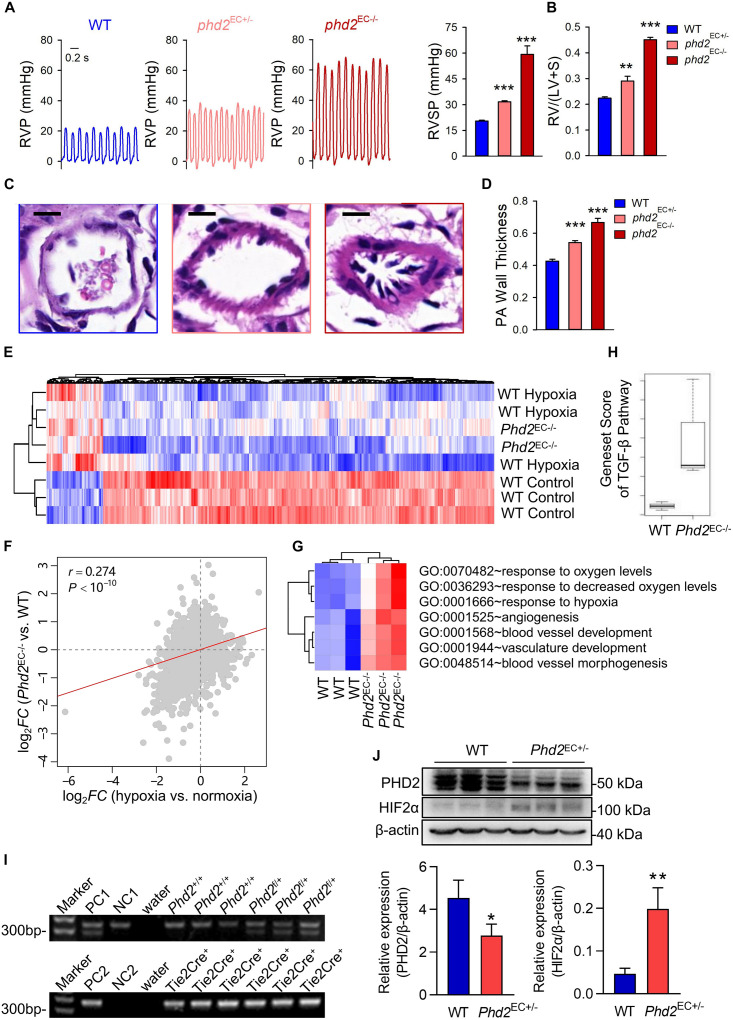
HIF2α-mediated GO terms involved in the development of *Phd2*-KO mediated spontaneous PH. **(A)** A representative waveform shows RVP (left) and statistical data (right) represents peak RVSP in WT, *Phd2*^*EC*+/–^ and *Phd2*^*EC*– /–^ mice (*n* = 6). Scale bars = 0.2 sec. **(B)** Statistical data of the RV/(LV + S) ratio shows RV hypertrophy defined by the Fulton index as a ratio [RV/(LV + S)] in WT, *Phd2*^*EC*+/–^ and *Phd2*^*EC*– /–^ mice (*n* = 6). **(C)** Typical *H & E* images of pulmonary arterioles from WT, *Phd2*^*EC*+/–^ and *Phd2*^*EC*– /–^ mice. Scale bars = 10 μm. **(D)** Statistical data shows PA wall thickness that generated by the ratio of wall area to total vessel area in PAs which were restricted less than 100 μm in diameter from WT, *Phd2*^*EC*+/–^ or *Phd2*^*EC*– /–^ mice (*n* = 6). **(E)** Expression heatmap of the genes commonly deregulated in both hypoxia and *Phd2*^*EC*– /–^ deficient groups. Red and blue represent relative up-regulation and down-regulation of gene expression, respectively. **(F)** Correlation in log_2_FC between hypoxia vs. normoxia mice (*X*-axis) and *Phd2*^*EC*– /–^ vs. WT mice (*Y*-axis) The correlation coefficient (r) and *P*-value were computed by Pearson correlation test. **(G)** Gene set score heatmap of the HIF2α-mediated GO biological process terms in WT and *Phd2*^*EC*– /–^ mice. In total, seven GO terms up-regulated in *Phd2*^*EC*– /–^ lungs were listed. Red and blue represent relative up-regulation and down-regulation of gene expression, respectively. **(H)** Comparison of gene set score of the TGF-β signaling pathway between WT and *Phd2*^*EC*– /–^ mice. **(I)** Genotyping of WT and *Phd2*^*EC*+/–^ mice. PC1: positive control 1 (*Phd2*^*f*/+^), PC2: positive control 2 (Tie2-Cre^+/–^), NC1: negative control 1 (*Phd2*^+/+^), NC2: negative control 2 (Tie2-Cre^– /–^). **(J)** Relative expression of the PHD2 and HIF2α in mice lung tissues detected by western blotting with β-actin as control (*n* = 3). Significance levels: *P < 0.05, **P < 0.01 and ***P < 0.001 (*t*-test).

To further investigate the correlation between HIF2α and the pattern of gene expression in a spontaneous PH model, endothelial cell-specific *Phd2* KO mice were generated. Both *Phd2*^*EC*+/–^ and *Phd2*^*EC*–/–^ mice developed spontaneous PH under normoxia. RVP representative tracings intuitively represented the changes in right ventricular pressure (Figure 4A). The right ventricular systolic pressure was 20.4869 ± 0.3781 mmHg in wild type mice (*Phd2*^*f*/f^), but 31.6415 ± 0.6030 and 59.2777 ± 4.5342 mmHg *in Phd2*^*EC*+/–^ and *Phd2*^*EC*–/–^ mice, respectively (Figure 4A). Elevated RVSP was also consistent with significantly aggravated RV hypertrophy in *Phd2*^*EC*–/–^ mice. The Fulton index of *Phd2*^*EC*+/–^ and *Phd2*^*EC*–/–^ mice, which was 0.2902 ± 0.0173 and 0.4507 ± 0.0086, significantly increased compared to *Phd2*^+/+^, respectively (Figure 4B). In *Phd2*^*EC*–/–^ mice, severe remodeling of the pulmonary arterioles characterized by a dramatic PA wall thickening was also observed (Figures 4C,D).

To investigate the molecular signaling events that promote PH development caused by *Phd2* knockout, we used gene microarray to analyze mRNA expression profiles in the lung tissues of *Phd2*^*EC*–/–^ mice. Globally, 459 genes (FDR < 10% and FC > 1.3) were identified which were universally deregulated in both hypoxia induced PH mice and *Phd2*^*EC*–/–^ mice (Figure 4E). The correlation in log_2_-transformed gene expression fold change (log_2_FC) between hypoxia vs. normoxic mice and *Phd2*^*EC*–/–^ vs. WT mice was derived at the whole-genome level. The two sets of fold changes showed a significant positive correlation (*Pearson* correlation test: *r* = 0.274, *P* < 10^–10^) (Figure 4F), demonstrating that the deregulated genes induced by hypoxia could be mimicked by *Phd2* Knockout. The gene set scores of the TGF-β signaling pathway (annotated by the KEGG database) in WT and *Phd2*^*EC*–/–^ mice were calculated. The result showed that the gene set score of the *Phd2*^*EC*–/–^ mice was significantly higher than that of WT mice (Figure 4H), which suggesting that the activation of the HIF2α and TGF-β signaling pathways in the *Phd2* deficiency mouse endothelial cells presumably resulting in severe pulmonary hypertension. Meanwhile, 7 of the 19 significantly down-regulated GO terms in *Hif2a*-KD cells were found to significantly (*t*-test: *P* < 0.05) overlap with the up-regulated GO gene sets in *Phd2*^*EC*–/–^ mice vs. WT (Figure 4G), We genotyped WT (*Phd2*^+/+^⋅Tie2-Cre^+/–^) and *Phd2*^*EC*+/–^ (*Phd2*^*f*/+^⋅Tie2-Cre^+/–^) mice (Figure 4I), and then WB assay was used to confirm the activation of HIF2α following partial deficiency of PHD2 in the mice lung tissues (Figure 4J). These findings reveal the opposing impact of HIF2α and PHD2 on PAH development.

## Discussion

PAH is a highly lethal vascular disease characterized by the medial remodeling in pulmonary arterioles and endothelial dysfunction, which leads to a progressive increase in PVR and pulmonary pressure (Rabinovitch, 2012). HIFs, especially HIF2α, has proved to play a significant role in the pathogenesis of PH. By deeply analyzing gene expression data from the GEO database, the present study was able to demonstrate that *Hif2a* knockdown regulates multiple biological processes associated with PAH, including but not limited to “blood vessel development” and “angiogenesis,” which were further shown to present among genes up-regulated in PAH patient lung tissues. More importantly, the 14 GO terms shared by *Hif2a* knockdown and up-regulated in PAH patient lung tissues show significantly better predictive power for PAH than random gene sets. Interestingly, 13 of the 19 down-regulated GO terms associated with *Hif2a* knockdown overlapped with the up-regulated GO terms in the peripheral blood mononuclear cells from PAH patients. By reason of the foregoing, these data indicate that HIF2α plays a critical role in PAH pathogenesis and HIF2α mediated gene sets can provide a distinctive and useful diagnostic method for distinguishing PAH human subjects. We investigated gene set expression profile rather than evaluating the expression level of individual genes for a compelling reason: mechanism-associated gene sets are highly reproducible and meaningful in transcriptome research (Abraham et al., 2010; Yang et al., 2012; Gardeux et al., 2014). Generally, single gene expression information is insufficient to unveil the underlying biological mechanisms. More importantly, most individual gene markers derived from genome-wide screening fail to be reproducible (Kern, 2012), because different genes jointly participating in a mechanism, such as a given GO term, may alternately be deregulated in different samples (Gardeux et al., 2014). Thus, the mechanistic gap between gene expression and transcriptome analysis can be effectively narrowed by mechanism-associated gene sets. Outliers appear in gene data sets of human CTR and PAH groups, which may be due to differences in their genetic background, or some of them have other underlying diseases. In clinical samples, it is often impossible to control intra group differences like animal models, which shows the advantages of animal model experiment.

HIF1α and HIF2α belong to a group of transcription factors, which regulate the transcription of most genes related to hypoxia adaptation (Hu et al., 2003). The degradation of HIFs depends on an oxygen dependent process. HIF1α and HIF2α are hydroxylated by PHDs, ubiquitinated and rapidly degraded through ubiquitin-proteasome under normoxia (Maxwell et al., 1999). However, under hypoxia, the decay of PHDs activity directly leads to the increase of HIF-α stability. This will facilitate their nuclear transposition, dimerization with ARNT and activation of their DNA-binding capacity. Then, the intracellular hypoxia adaptive regulation will be lunched (Hu et al., 2003). Some evidences show that HIF2α can act as an independent pathogenic factor during PAH. A mutation prevents HIF2α hydroxylation by PHDs, which accumulates HIF2α that will ultimately contribute to the development of PAH both in mice and human (Brusselmans et al., 2003; Tan et al., 2013). Current studies have shown that PHD2 expression was reduced in endothelial cells at the lesion sites of the pulmonary arteries in idiopathic PAH patients, and the loss of PHD2 in endothelial cells activates HIF2α rather than HIF1α (Dai et al., 2016). This is the reason that *Phd2* KO mice lung tissues were chosen for experimental verification. These studies indicate the importance of the PHD2/HIF2α axis in PAH development.

Hypoxia-inducible genes been transcriptional activated include vascular endothelial growth factor-a (VEGF-A), erythropoietin (EPO) and inducible nitric oxide synthase (iNOS) (Semenza and Wang, 1992; Melillo et al., 1995; Tuder et al., 1995). The adaptation to hypoxia mediated by HIFs is mainly achieved by regulating the protein content of α subunit. A hypoxic environment is a common occurrence in the lung in the pathological situation of PAH. As HIF-α signaling contributes to the endothelial dysfunction, vascular smooth muscle cells (VSMCs) proliferation, migration and tissue remodeling processes, it has the potential to become a therapeutic target.

HIF1α is widely expressed in the cells of most mammals, while HIF2α is restricted and highly expressed in the endothelial cells (Patel and Simon, 2008). The accumulation of HIF2α in ECs leads to increased ET-1 production, enhanced HIF1α activity in PASMCs during PAH, and the HIF target genes up-regulation (Shimoda and Laurie, 2014). It has been reported that *Hif2a*^+/–^ mice exhibit alleviated PAH pathological symptoms, including reduced right ventricular pressure and vascular remodeling (Brusselmans et al., 2003). The result of a statistical study showed that there is a correlation between HIF2α dysfunction and the decreased PAP in Tibetan natives (van Patot and Gassmann, 2011). Conversely, HIF2α overexpression is associated with development of PH in both humans (Gale et al., 2008; Formenti et al., 2011) and mice (Tan et al., 2013). Our GO analysis of PAH patient lung tissues samples further strengthens the important role of HIF2α in PAH. Comparing to the controls, 14 up-regulated GO gene sets (*t*-test: *P* < 0.05) in PAH patients overlapped with the down-regulated GO gene sets in *Hif2a*-KD cells (Figures 1, 2). As we can see in Figure 2, most of the GO terms up-regulated in PAH patient samples are associated with the function of HIF2α. PAH is a disease related to the dysregulation of the lung vascular system and we also identified the up-regulation of GO gene sets related to angiogenesis and blood vessel development (GO: 0001525, GO: 0045766, GO: 0001568). HIF2α has been shown to control genes involved in the regulation of angiogenesis and vascular development, and we identified two such gene among the genes significantly down-regulated in *Hif2a*-KD cells: vascular endothelial growth factor A (VEGFA) and sirtuin 1 (SIRT1). By binding its receptor VEGFR2, VEGFA regulates angiogenesis and vascular permeability through promoting the proliferation and survival of ECs, where the downstream signaling pathways include phosphoinositide 3 kinase (PI3K)/Akt, focal adhesion kinase (FAK), Rho family GTPases, nitric oxide (NO), and p38 mitogen−activated protein kinase (MAPK) (Claesson-Welsh and Welsh, 2013). Moreover, a recent study showed HIV protein transactivator of transcription (TAT) stimulated the proliferation of pulmonary artery SMCs (PASMCs) through increasing the expression VEGFA, and may be a potential therapeutic target for the treatment of HIV-associated PAH (Guo et al., 2018). SIRT1 is a NAD^+^-dependent deacetylase shown to play important roles in angiogenesis signaling both *in vitro* and in mice through controlling the acetylation of the forkhead transcription factor, Foxo1, which is highly expressed in ECs and mediates the expression of angiopoietin 2 (Ang2) (Potente et al., 2005, 2007). In addition, SIRT1 also regulates the proliferation of PASMCs by activating peroxisome proliferator-activated receptor gamma coactivator 1-alpha (PGC-1α) (Zurlo et al., 2018). Thus, it is likely that uncontrolled activation of HIF2α induces the overexpression of its downstream genes, such as VEGFA and SIRT1, which causes dysregulated development of the lung vascular system and contributes to the development of PAH.

In conclusion, by deeply analyzing selected gene expression data deposited in the GEO database we were able to demonstrate that HIF2α-regulated gene sets significantly overlapped with gene sets up-regulated in PAH patient lung tissue samples and peripheral blood mononuclear cells. HIF2α, as a key regulatory molecule, plays a significant role in the pathogenesis of PAH and speculate that this methodology could provide a new application prospect in identifying PAH patients.

## Data Availability Statement

The raw data supporting the conclusions of this article will be made available by the authors, without undue reservation.

## Ethics Statement

The animal study was reviewed and approved by the Institutional Animal Care and Use Committee (IACUC) of Guangzhou Medical University.

## Author Contributions

HT designed the project. JZ, LZ, YH, GC, and CB performed the experiments. YH, TZ, and HT wrote the manuscript with input from JW, WL, and SB. All authors contributed to the article and approved the submitted version.

## Conflict of Interest

The authors declare that the research was conducted in the absence of any commercial or financial relationships that could be construed as a potential conflict of interest.

## Publisher’s Note

All claims expressed in this article are solely those of the authors and do not necessarily represent those of their affiliated organizations, or those of the publisher, the editors and the reviewers. Any product that may be evaluated in this article, or claim that may be made by its manufacturer, is not guaranteed or endorsed by the publisher.
