# Supplementary material for: Hypoxia-Inducible Factor 2-Alpha Mediated Gene Sets Differentiate Pulmonary Arterial Hypertension
Source: Front Cell Dev Biol. 2021 Aug 5;9:701247. doi: 10.3389/fcell.2021.701247 (PMC8375387; doi:10.3389/fcell.2021.701247)
Supplement: Supplementary file 1 [file Table_1.DOCX]

**Supplementary Figure S1.** Volcano plot indicates the differential expression genes in the lung tissues of IPAH patients and controls. The volcano plot was generated online at https://www.ncbi.nlm.nih.gov/geo

Supplementary Table S1. The genes upregulated in *Hif2a*-KD VHL-deficient human ccRCC cells.

| Gene symbol | Gene title | FC | FDR (%) |
| --- | --- | --- | --- |
| *ARSJ* | arylsulfatase family, member J | 1.3921 | 0.00 |
| *TAF13* | TAF13 RNA polymerase II, TATA box binding protein (TBP)-associated factor, 18kDa | 1.3626 | 0.00 |
| *ASIC2* | acid-sensing (proton-gated) ion channel 2 | 1.5207 | 0.00 |
| *MTSS1L* | metastasis suppressor 1-like | 1.375 | 0.00 |
| *TMEM30A* | transmembrane protein 30A | 1.5733 | 0.00 |
| *CLDN1* | claudin 1 | 1.4405 | 0.00 |
| *POLH* | polymerase (DNA directed), eta | 1.5261 | 0.00 |
| *STOM* | stomatin | 1.3967 | 0.00 |
| *CPE* | carboxypeptidase E | 1.4791 | 0.00 |
| *MAP3K2* | mitogen-activated protein kinase kinase kinase 2 | 1.3015 | 0.00 |
| *TMEM2* | transmembrane protein 2 | 1.3918 | 0.00 |
| *AK1* | adenylate kinase 1 | 1.3063 | 0.00 |
| *SIPA1L2* | signal-induced proliferation-associated 1 like 2 | 1.3873 | 0.00 |
| *ADCY9* | adenylate cyclase 9 | 1.337 | 0.00 |
| *KIAA1147* | KIAA1147 | 1.376 | 0.00 |
| *RSPO3* | R-spondin 3 | 1.3197 | 0.00 |
| *TM4SF1* | transmembrane 4 L six family member 1 | 1.4397 | 0.00 |
| *IFT27* | intraflagellar transport 27 homolog (Chlamydomonas) | 1.4997 | 0.00 |
| *SDC4* | syndecan 4 | 1.6597 | 0.00 |
| *EXPH5* | exophilin 5 | 1.7295 | 0.00 |
| *DCP2* | decapping mRNA 2 | 1.841 | 0.00 |
| *C9orf78* | chromosome 9 open reading frame 78 | 1.5742 | 0.00 |
| *NOLC1* | nucleolar and coiled-body phosphoprotein 1 | 1.5715 | 0.00 |
| *NTN4* | netrin 4 | 2.0714 | 0.00 |
| *MON2* | MON2 homolog (S. cerevisiae) | 1.3172 | 0.00 |
| *ZW10* | zw10 kinetochore protein | 1.5467 | 0.00 |
| *VPS4A* | vacuolar protein sorting 4 homolog A (S. cerevisiae) | 1.3983 | 0.00 |
| *TM7SF3* | transmembrane 7 superfamily member 3 | 1.3637 | 0.00 |
| *SESN1* | sestrin 1 | 1.7833 | 0.00 |
| *SFN* | stratifin | 1.6833 | 0.00 |
| *ANKRD1* | ankyrin repeat domain 1 (cardiac muscle) | 2.1343 | 0.00 |
| *BTG2* | BTG family, member 2 | 1.882 | 0.00 |
| *SBDS* | Shwachman-Bodian-Diamond syndrome | 1.4076 | 0.00 |
| *MTO1* | mitochondrial tRNA translation optimization 1 | 1.3407 | 0.00 |
| *EPHB4* | EPH receptor B4 | 1.3633 | 0.00 |
| *THSD1* | thrombospondin, type I, domain containing 1 | 1.4842 | 0.00 |
| NCEH1 | neutral cholesterol ester hydrolase 1 | 1.3355 | 0.00 |
| *ANKRD52* | ankyrin repeat domain 52 | 1.4137 | 0.00 |
| *FLNB* | filamin B, beta | 1.33 | 0.00 |
| *SETD7* | SET domain containing (lysine methyltransferase) 7 | 1.6077 | 0.00 |
| *SLC37A3* | solute carrier family 37 (glycerol-3-phosphate transporter), member 3 | 1.4123 | 0.00 |
| *PRPF3* | pre-mRNA processing factor 3 | 1.3068 | 0.00 |
| *OGFR* | opioid growth factor receptor | 1.4081 | 0.00 |
| *PLEKHB2* | pleckstrin homology domain containing, family B (evectins) member 2 | 1.3973 | 0.00 |
| *UBR3* | ubiquitin protein ligase E3 component n-recognin 3 (putative) | 1.4746 | 0.00 |
| *CASP7* | caspase 7, apoptosis-related cysteine peptidase | 1.4497 | 0.00 |
| *RGS19* | regulator of G-protein signaling 19 | 1.3606 | 0.00 |
| *FAM126B* | family with sequence similarity 126, member B | 1.6207 | 0.00 |
| *G3BP2* | GTPase activating protein (SH3 domain) binding protein 2 | 1.4296 | 0.00 |
| *RAPGEF1* | Rap guanine nucleotide exchange factor (GEF) 1 | 1.3006 | 0.00 |
| *ZHX1* | zinc fingers and homeoboxes 1 | 1.3249 | 0.00 |
| *TMTC3* | transmembrane and tetratricopeptide repeat containing 3 | 1.3809 | 0.00 |
| *HSPB8* | heat shock 22kDa protein 8 | 1.3331 | 0.00 |
| *PPP1R9B* | protein phosphatase 1, regulatory subunit 9B | 1.3491 | 0.00 |
| *FDXR* | ferredoxin reductase | 1.4805 | 0.00 |
| *STAT3* | signal transducer and activator of transcription 3 (acute-phase response factor) | 1.4586 | 0.00 |
| *UBE4A* | ubiquitination factor E4A | 1.3207 | 0.00 |
| *TRAPPC6B* | trafficking protein particle complex 6B | 1.3288 | 0.00 |
| *WDR63* | WD repeat domain 63 | 1.6576 | 0.00 |
| *POLR2D* | polymerase (RNA) II (DNA directed) polypeptide D | 1.3077 | 0.00 |
| *HRAS* | v-Ha-ras Harvey rat sarcoma viral oncogene homolog | 1.3137 | 0.00 |
| *RALGAPB* | Ral GTPase activating protein, beta subunit (non-catalytic) | 1.3629 | 0.00 |
| *HCFC2* | host cell factor C2 | 1.3878 | 0.00 |
| *MCCC1* | methylcrotonoyl-CoA carboxylase 1 (alpha) | 1.31 | 0.00 |
| *HEXIM1* | hexamethylene bis-acetamide inducible 1 | 1.3561 | 0.00 |
| *PPME1* | protein phosphatase methylesterase 1 | 1.3228 | 0.00 |
| *KLHDC3* | kelch domain containing 3 | 1.4103 | 0.00 |
| *BICD2* | bicaudal D homolog 2 (Drosophila) | 1.4924 | 0.00 |
| *VPS45* | vacuolar protein sorting 45 homolog (S. cerevisiae) | 1.3342 | 0.00 |
| *SHISA3* | shisa homolog 3 (Xenopus laevis) | 1.5191 | 0.00 |
| *FAM3C* | family with sequence similarity 3, member C | 1.3102 | 0.00 |
| *HSPA4L* | heat shock 70kDa protein 4-like | 1.3544 | 0.00 |
| *ZMAT3* | zinc finger, matrin-type 3 | 1.546 | 0.00 |
| *PLCD3* | phospholipase C, delta 3 | 1.3569 | 0.00 |
| *DUS1L* | dihydrouridine synthase 1-like (S. cerevisiae) | 1.3369 | 0.00 |
| *KLHDC8B* | kelch domain containing 8B | 1.4094 | 0.00 |
| *STX12* | syntaxin 12 | 1.8608 | 0.00 |
| MKNK2 | MAP kinase interacting serine/threonine kinase 2 | 1.395 | 0.00 |
| *MAP2K4* | mitogen-activated protein kinase kinase 4 | 1.3483 | 0.00 |
| *DUSP14* | dual specificity phosphatase 14 | 1.6006 | 0.00 |
| *ZDHHC18* | zinc finger, DHHC-type containing 18 | 1.4935 | 0.00 |
| *PPM1D* | protein phosphatase, Mg2+/Mn2+ dependent, 1D | 1.6536 | 0.00 |
| *MFN1* | mitofusin 1 | 1.3141 | 0.00 |
| *TMEM81* | transmembrane protein 81 | 1.3747 | 0.00 |
| *R3HCC1* | R3H domain and coiled-coil containing 1 | 1.4126 | 0.00 |
| *PEX12* | peroxisomal biogenesis factor 12 | 1.3124 | 0.00 |
| *SULF2* | sulfatase 2 | 1.5616 | 0.00 |
| *SKA2* | spindle and kinetochore associated complex subunit 2 | 1.3206 | 0.15 |
| *KAT2B* | K(lysine) acetyltransferase 2B | 1.3594 | 0.15 |
| *KIF1B* | kinesin family member 1B | 1.3266 | 0.15 |
| *SLC25A20* | solute carrier family 25 (carnitine/acylcarnitine translocase), member 20 | 1.5335 | 0.15 |
| *SEMA6B* | sema domain, transmembrane domain (TM), and cytoplasmic domain, (semaphorin) 6B | 1.3006 | 0.15 |
| *CHKA* | choline kinase alpha | 1.4492 | 0.15 |
| *DNAJB4* | DnaJ (Hsp40) homolog, subfamily B, member 4 | 1.3299 | 0.15 |
| *KIF1C* | kinesin family member 1C | 1.4039 | 0.15 |
| *GK5* | glycerol kinase 5 (putative) | 1.4319 | 0.15 |
| *POSTN* | periostin, osteoblast specific factor | 1.342 | 0.15 |
| *EPHA2* | EPH receptor A2 | 1.4226 | 0.15 |
| *CHORDC1* | cysteine and histidine-rich domain (CHORD) containing 1 | 1.3856 | 0.15 |
| *ANKRA2* | ankyrin repeat, family A (RFXANK-like), 2 | 1.4423 | 0.15 |
| *GBP2* | guanylate binding protein 2, interferon-inducible | 1.5 | 0.15 |
| *TBC1D2* | TBC1 domain family, member 2 | 1.4454 | 0.15 |
| *ATP5L* | ATP synthase, H+ transporting, mitochondrial Fo complex, subunit G | 1.372 | 0.15 |
| *FAM35A* | family with sequence similarity 35, member A | 1.3796 | 0.15 |
| *CAB39* | calcium binding protein 39 | 1.3153 | 0.19 |
| *XPC* | xeroderma pigmentosum, complementation group C | 1.427 | 0.19 |
| *DDX50* | DEAD (Asp-Glu-Ala-Asp) box polypeptide 50 | 1.3119 | 0.19 |
| *NUDCD3* | NudC domain containing 3 | 1.4309 | 0.19 |
| *GPC1* | glypican 1 | 1.37 | 0.19 |
| *ME1* | malic enzyme 1, NADP(+)-dependent, cytosolic | 1.3407 | 0.19 |
| *CYR61* | cysteine-rich, angiogenic inducer, 61 | 1.4023 | 0.19 |
| *RIOK2* | RIO kinase 2 | 1.3622 | 0.19 |
| *CDIP1* | cell death-inducing p53 target 1 | 1.3089 | 0.19 |
| *FNIP2* | folliculin interacting protein 2 | 1.4171 | 0.19 |
| *ATF1* | activating transcription factor 1 | 1.4585 | 0.19 |
| *ZNF585B* | zinc finger protein 585B | 1.3787 | 0.19 |
| *ZNF382* | zinc finger protein 382 | 1.3309 | 0.19 |
| *AK3* | adenylate kinase 3 | 1.3209 | 0.19 |
| *TRAF4* | TNF receptor-associated factor 4 | 1.3166 | 0.19 |
| *DZIP3* | DAZ interacting zinc finger protein 3 | 1.3799 | 0.19 |
| *CYFIP2* | cytoplasmic FMR1 interacting protein 2 | 1.4464 | 0.19 |
| *THBS1* | thrombospondin 1 | 1.366 | 0.19 |
| *B4GALT1* | UDP-Gal:betaGlcNAc beta 1,4- galactosyltransferase, polypeptide 1 | 1.488 | 0.19 |
| *CCP110* | centriolar coiled coil protein 110kDa | 1.3107 | 0.19 |
| *PRDM1* | PR domain containing 1, with ZNF domain | 2.0499 | 0.19 |
| *SNAPC1* | small nuclear RNA activating complex, polypeptide 1, 43kDa | 1.3547 | 0.19 |
| *RASGRP3* | RAS guanyl releasing protein 3 (calcium and DAG-regulated) | 1.5193 | 0.19 |
| *DENND5A* | DENN/MADD domain containing 5A | 1.3276 | 0.19 |
| *AHNAK2* | AHNAK nucleoprotein 2 | 1.3055 | 0.19 |
| *NRG1* | neuregulin 1 | 1.3294 | 0.19 |
| *GOLGA4* | golgin A4 | 1.361 | 0.19 |
| *GANAB* | glucosidase, alpha; neutral AB | 1.4092 | 0.19 |
| *PCNXL3* | pecanex-like 3 (Drosophila) | 1.4189 | 0.19 |
| *ACTA2* | actin, alpha 2, smooth muscle, aorta | 1.5163 | 0.19 |
| *STK17A* | serine/threonine kinase 17a | 1.3112 | 0.19 |
| *VAMP4* | vesicle-associated membrane protein 4 | 1.3529 | 0.19 |
| *ABCA12* | ATP-binding cassette, sub-family A (ABC1), member 12 | 1.767 | 0.19 |
| *INSIG2* | insulin induced gene 2 | 1.3683 | 0.19 |
| *CLTB* | clathrin, light chain B | 1.3366 | 0.29 |
| *RNF19B* | ring finger protein 19B | 1.4933 | 0.29 |
| *HSDL2* | hydroxysteroid dehydrogenase like 2 | 1.3975 | 0.29 |
| *CCNA1* | cyclin A1 | 1.4899 | 0.29 |
| *TAF3* | TAF3 RNA polymerase II, TATA box binding protein (TBP)-associated factor, 140kDa | 1.3134 | 0.29 |
| *ASCC2* | activating signal cointegrator 1 complex subunit 2 | 1.3582 | 0.29 |
| *ZZEF1* | zinc finger, ZZ-type with EF-hand domain 1 | 1.3415 | 0.29 |
| *ZNF658* | zinc finger protein 658 | 1.4006 | 0.29 |
| *ZNF680* | zinc finger protein 680 | 1.3966 | 0.29 |
| *FBXO32* | F-box protein 32 | 1.3124 | 0.29 |
| *BACE1* | beta-site APP-cleaving enzyme 1 | 1.4061 | 0.29 |
| *IL18* | interleukin 18 (interferon-gamma-inducing factor) | 1.337 | 0.29 |
| *IER2* | immediate early response 2 | 1.3021 | 0.29 |
| *GREB1L* | growth regulation by estrogen in breast cancer-like | 1.3288 | 0.37 |
| *PRKAB1* | protein kinase, AMP-activated, beta 1 non-catalytic subunit | 1.383 | 0.37 |
| *CTGF* | connective tissue growth factor | 1.309 | 0.37 |
| *PTBP2* | polypyrimidine tract binding protein 2 | 1.437 | 0.37 |
| *TP53I3* | tumor protein p53 inducible protein 3 | 1.8091 | 0.37 |
| *HABP4* | hyaluronan binding protein 4 | 1.3782 | 0.37 |
| *MAMDC2* | MAM domain containing 2 | 1.3469 | 0.37 |
| *DGKA* | diacylglycerol kinase, alpha 80kDa | 1.3859 | 0.37 |
| *USP15* | ubiquitin specific peptidase 15 | 1.3172 | 0.37 |
| *DEXI* | Dexi homolog (mouse) | 1.358 | 0.37 |
| *ZCCHC10* | zinc finger, CCHC domain containing 10 | 1.304 | 0.37 |
| *SLC12A4* | solute carrier family 12 (potassium/chloride transporters), member 4 | 1.3287 | 0.37 |
| *PLK3* | polo-like kinase 3 | 1.3819 | 0.37 |
| *CABYR* | calcium binding tyrosine-(Y)-phosphorylation regulated | 1.3185 | 0.37 |
| *ARHGAP18* | Rho GTPase activating protein 18 | 1.3874 | 0.37 |
| *RPL41* | ribosomal protein L41 | 1.4395 | 0.37 |
| *GLE1* | GLE1 RNA export mediator homolog (yeast) | 1.3326 | 0.56 |
| *INPP5D* | inositol polyphosphate-5-phosphatase, 145kDa | 1.556 | 0.56 |
| *PKP2* | plakophilin 2 | 1.3622 | 0.56 |
| *CENPW* | centromere protein W | 1.3626 | 0.56 |
| *SCFD2* | sec1 family domain containing 2 | 1.3027 | 0.56 |
| *ADCK3* | aarF domain containing kinase 3 | 1.314 | 0.56 |
| *GPR87* | G protein-coupled receptor 87 | 1.5028 | 0.56 |
| *PDE5A* | phosphodiesterase 5A, cGMP-specific | 1.3717 | 0.56 |
| *IPPK* | inositol 1,3,4,5,6-pentakisphosphate 2-kinase | 1.3519 | 0.56 |
| *SLC46A3* | solute carrier family 46, member 3 | 1.4009 | 0.74 |
| *KLF4* | Kruppel-like factor 4 (gut) | 1.3379 | 0.74 |
| *LACC1* | laccase (multicopper oxidoreductase) domain containing 1 | 1.4279 | 0.74 |
| *CKS2* | CDC28 protein kinase regulatory subunit 2 | 1.3005 | 0.74 |
| *EPDR1* | ependymin related protein 1 (zebrafish) | 1.302 | 0.74 |
| *ZNF267* | zinc finger protein 267 | 1.3515 | 0.74 |
| *SLIT2* | slit homolog 2 (Drosophila) | 1.4205 | 0.74 |
| *CDK14* | cyclin-dependent kinase 14 | 1.4111 | 0.74 |
| *SPATA18* | spermatogenesis associated 18 | 1.3059 | 0.74 |
| *LATS2* | large tumor suppressor kinase 2 | 1.3804 | 0.74 |
| *RAB19* | RAB19, member RAS oncogene family | 1.3374 | 0.74 |
| *ISCU* | iron-sulfur cluster scaffold homolog (E. coli) | 1.3027 | 0.94 |
| *FAS* | Fas cell surface death receptor | 1.3263 | 0.94 |
| *IFFO2* | intermediate filament family orphan 2 | 1.3797 | 0.94 |
| *APAF1* | apoptotic peptidase activating factor 1 | 1.3141 | 0.94 |
| *PLA2G12A* | phospholipase A2, group XIIA | 1.3237 | 0.94 |
| *ZNF654* | zinc finger protein 654 | 1.5524 | 0.94 |

Supplementary Table S2. The genes downregulated in *Hif2a*-KD VHL-deficient human ccRCC cells.

| Gene symbol | Gene title | FC | FDR (%) |
| --- | --- | --- | --- |
| *SLC35D1* | solute carrier family 35 (UDP-glucuronic acid/UDP-N-acetylgalactosamine dual transporter), member D1 | 0.5661 | 0.00 |
| *FAM65A* | family with sequence similarity 65, member A | 0.6404 | 0.00 |
| *GTF2E1* | general transcription factor IIE, polypeptide 1, alpha 56kDa | 0.633 | 0.00 |
| *TMEM52B* | transmembrane protein 52B | 0.5654 | 0.00 |
| *HMOX1* | heme oxygenase (decycling) 1 | 0.5579 | 0.00 |
| *STC1* | stanniocalcin 1 | 0.537 | 0.00 |
| *PLAT* | plasminogen activator, tissue | 0.5299 | 0.00 |
| *TGFBR2* | transforming growth factor, beta receptor II (70/80kDa) | 0.5616 | 0.00 |
| *APOL1* | apolipoprotein L, 1 | 0.6057 | 0.00 |
| *EPAS1* | endothelial PAS domain protein 1 | 0.2853 | 0.00 |
| *CITED2* | Cbp/p300-interacting transactivator, with Glu/Asp-rich carboxy-terminal domain, 2 | 0.6937 | 0.00 |
| *PKIA* | protein kinase (cAMP-dependent, catalytic) inhibitor alpha | 0.5004 | 0.00 |
| *SEC14L2* | SEC14-like 2 (S. cerevisiae) | 0.7099 | 0.00 |
| *DNAJC16* | DnaJ (Hsp40) homolog, subfamily C, member 16 | 0.6896 | 0.00 |
| *ARRDC3* | arrestin domain containing 3 | 0.6098 | 0.00 |
| *TMEM14A* | transmembrane protein 14A | 0.6503 | 0.00 |
| *BNIP3L* | BCL2/adenovirus E1B 19kDa interacting protein 3-like | 0.6027 | 0.00 |
| *SEMA4B* | sema domain, immunoglobulin domain (Ig), transmembrane domain (TM) and short cytoplasmic domain, (semaphorin) 4B | 0.7196 | 0.00 |
| *CPT1A* | carnitine palmitoyltransferase 1A (liver) | 0.7382 | 0.00 |
| *CTDSP2* | CTD (carboxy-terminal domain, RNA polymerase II, polypeptide A) small phosphatase 2 | 0.6012 | 0.00 |
| *ITGAX* | integrin, alpha X (complement component 3 receptor 4 subunit) | 0.7071 | 0.00 |
| *MAP2K6* | mitogen-activated protein kinase kinase 6 | 0.5556 | 0.00 |
| *BRPF3* | bromodomain and PHD finger containing, 3 | 0.7347 | 0.00 |
| *C5orf22* | chromosome 5 open reading frame 22 | 0.6669 | 0.00 |
| *CDA* | cytidine deaminase | 0.6913 | 0.00 |
| *ASB13* | ankyrin repeat and SOCS box containing 13 | 0.6663 | 0.00 |
| *PLSCR4* | phospholipid scramblase 4 | 0.6076 | 0.00 |
| *ZBTB41* | zinc finger and BTB domain containing 41 | 0.6759 | 0.00 |
| *PNMA2* | paraneoplastic Ma antigen 2 | 0.7167 | 0.00 |
| *LIPA* | lipase A, lysosomal acid, cholesterol esterase | 0.4847 | 0.00 |
| *KIAA1191* | KIAA1191 | 0.5848 | 0.00 |
| *GRAMD3* | GRAM domain containing 3 | 0.6434 | 0.00 |
| *MBNL2* | muscleblind-like splicing regulator 2 | 0.6727 | 0.00 |
| *LOXL2* | lysyl oxidase-like 2 | 0.7289 | 0.00 |
| *EML4* | echinoderm microtubule associated protein like 4 | 0.6426 | 0.00 |
| *MAD2L2* | MAD2 mitotic arrest deficient-like 2 (yeast) | 0.6824 | 0.00 |
| *TGFBI* | transforming growth factor, beta-induced, 68kDa | 0.6198 | 0.00 |
| *MFF* | mitochondrial fission factor | 0.7098 | 0.00 |
| *ITPR1* | inositol 1,4,5-trisphosphate receptor, type 1 | 0.4997 | 0.00 |
| *ADM* | adrenomedullin | 0.6776 | 0.00 |
| *SLC43A3* | solute carrier family 43, member 3 | 0.6796 | 0.00 |
| *ISOC1* | isochorismatase domain containing 1 | 0.5135 | 0.00 |
| *C1QL1* | complement component 1, q subcomponent-like 1 | 0.7287 | 0.00 |
| *LRRC49* | leucine rich repeat containing 49 | 0.7024 | 0.00 |
| *WDR91* | WD repeat domain 91 | 0.6967 | 0.00 |
| *NAV1* | neuron navigator 1 | 0.6571 | 0.00 |
| *SYT3* | synaptotagmin III | 0.7655 | 0.00 |
| *SLC25A32* | solute carrier family 25 (mitochondrial folate carrier), member 32 | 0.5806 | 0.00 |
| *PCBD1* | pterin-4 alpha-carbinolamine dehydratase/dimerization cofactor of hepatocyte nuclear factor 1 alpha | 0.5771 | 0.00 |
| *TRAM2* | translocation associated membrane protein 2 | 0.6011 | 0.00 |
| *CHST11* | carbohydrate (chondroitin 4) sulfotransferase 11 | 0.5862 | 0.00 |
| *CCNDBP1* | cyclin D-type binding-protein 1 | 0.6846 | 0.00 |
| *G3BP1* | GTPase activating protein (SH3 domain) binding protein 1 | 0.6896 | 0.00 |
| *ANKFY1* | ankyrin repeat and FYVE domain containing 1 | 0.687 | 0.00 |
| *CYB5R2* | cytochrome b5 reductase 2 | 0.4421 | 0.00 |
| *EIF4EBP2* | eukaryotic translation initiation factor 4E binding protein 2 | 0.4399 | 0.00 |
| *REEP3* | receptor accessory protein 3 | 0.5876 | 0.00 |
| *GOLGA7* | golgin A7 | 0.6619 | 0.00 |
| *LSM12* | LSM12 homolog (S. cerevisiae) | 0.682 | 0.00 |
| *TOR1AIP1* | torsin A interacting protein 1 | 0.7377 | 0.00 |
| *EGLN3* | egl nine homolog 3 (C. elegans) | 0.6355 | 0.00 |
| *NID1* | nidogen 1 | 0.6278 | 0.00 |
| *TMEM64* | transmembrane protein 64 | 0.6098 | 0.00 |
| *IGSF3* | immunoglobulin superfamily, member 3 | 0.6689 | 0.00 |
| *TMEM62* | transmembrane protein 62 | 0.5276 | 0.00 |
| *HIP1* | huntingtin interacting protein 1 | 0.6415 | 0.00 |
| *UCP2* | uncoupling protein 2 (mitochondrial, proton carrier) | 0.717 | 0.00 |
| *TCP11L2* | t-complex 11, testis-specific-like 2 | 0.6442 | 0.00 |
| *CTTNBP2NL* | CTTNBP2 N-terminal like | 0.6922 | 0.00 |
| *IL6ST* | interleukin 6 signal transducer (gp130, oncostatin M receptor) | 0.7241 | 0.00 |
| *RIPK1* | receptor (TNFRSF)-interacting serine-threonine kinase 1 | 0.7386 | 0.00 |
| *BAG2* | BCL2-associated athanogene 2 | 0.6877 | 0.00 |
| *SPAST* | spastin | 0.6377 | 0.00 |
| *STRN4* | striatin, calmodulin binding protein 4 | 0.6903 | 0.00 |
| *FAM117B* | family with sequence similarity 117, member B | 0.75 | 0.00 |
| *NUP160* | nucleoporin 160kDa | 0.6448 | 0.00 |
| *PREX2* | phosphatidylinositol-3,4,5-trisphosphate-dependent Rac exchange factor 2 | 0.5359 | 0.00 |
| *TRERF1* | transcriptional regulating factor 1 | 0.6535 | 0.00 |
| *SMARCE1* | SWI/SNF related, matrix associated, actin dependent regulator of chromatin, subfamily e, member 1 | 0.6158 | 0.00 |
| *RASSF2* | Ras association (RalGDS/AF-6) domain family member 2 | 0.54 | 0.00 |
| *PTBP3* | polypyrimidine tract binding protein 3 | 0.4806 | 0.00 |
| *NDRG1* | N-myc downstream regulated 1 | 0.4927 | 0.00 |
| *ATP6V0D2* | ATPase, H+ transporting, lysosomal 38kDa, V0 subunit d2 | 0.607 | 0.00 |
| *NRAS* | neuroblastoma RAS viral (v-ras) oncogene homolog | 0.646 | 0.00 |
| *MYH9* | myosin, heavy chain 9, non-muscle | 0.707 | 0.00 |
| *GRAMD1A* | GRAM domain containing 1A | 0.7341 | 0.00 |
| *NAPG* | N-ethylmaleimide-sensitive factor attachment protein, gamma | 0.7666 | 0.00 |
| *NCR3LG1* | natural killer cell cytotoxicity receptor 3 ligand 1 | 0.6771 | 0.00 |
| *CDH11* | cadherin 11, type 2, OB-cadherin (osteoblast) | 0.686 | 0.00 |
| *ENO2* | enolase 2 (gamma, neuronal) | 0.6709 | 0.00 |
| *VEGFA* | vascular endothelial growth factor A | 0.6142 | 0.00 |
| *ITGB3* | integrin, beta 3 (platelet glycoprotein IIIa, antigen CD61) | 0.6499 | 0.00 |
| *CACNB3* | calcium channel, voltage-dependent, beta 3 subunit | 0.7333 | 0.00 |
| *LEF1* | lymphoid enhancer-binding factor 1 | 0.6811 | 0.00 |
| *DUSP18* | dual specificity phosphatase 18 | 0.5913 | 0.00 |
| *TNFAIP6* | tumor necrosis factor, alpha-induced protein 6 | 0.5268 | 0.00 |
| *PRUNE* | prune exopolyphosphatase | 0.7596 | 0.00 |
| *RARB* | retinoic acid receptor, beta | 0.719 | 0.00 |
| *TTLL6* | tubulin tyrosine ligase-like family, member 6 | 0.7111 | 0.00 |
| *DAZAP2* | DAZ associated protein 2 | 0.6584 | 0.00 |
| *RAB8B* | RAB8B, member RAS oncogene family | 0.7475 | 0.00 |
| *MUC13* | mucin 13, cell surface associated | 0.5813 | 0.00 |
| *HSD17B4* | hydroxysteroid (17-beta) dehydrogenase 4 | 0.6754 | 0.00 |
| *SLC1A1* | solute carrier family 1 (neuronal/epithelial high affinity glutamate transporter, system Xag), member 1 | 0.7139 | 0.00 |
| *TFAM* | transcription factor A, mitochondrial | 0.7161 | 0.00 |
| *CDKN2AIPNL* | CDKN2A interacting protein N-terminal like | 0.6779 | 0.00 |
| *IRS2* | insulin receptor substrate 2 | 0.7418 | 0.00 |
| *ZNHIT1* | zinc finger, HIT-type containing 1 | 0.7191 | 0.00 |
| *NIPSNAP3A* | nipsnap homolog 3A (C. elegans) | 0.7342 | 0.00 |
| *PPP2R1B* | protein phosphatase 2, regulatory subunit A, beta | 0.7018 | 0.00 |
| *SLC25A24* | solute carrier family 25 (mitochondrial carrier; phosphate carrier), member 24 | 0.7041 | 0.00 |
| *BEND7* | BEN domain containing 7 | 0.7475 | 0.00 |
| *ASAP1* | ArfGAP with SH3 domain, ankyrin repeat and PH domain 1 | 0.7318 | 0.00 |
| *CAMK2N1* | calcium/calmodulin-dependent protein kinase II inhibitor 1 | 0.6505 | 0.00 |
| *TMEM171* | transmembrane protein 171 | 0.6962 | 0.00 |
| *PRR5L* | proline rich 5 like | 0.6973 | 0.00 |
| *HIST1H2BH* | histone cluster 1, H2bh | 0.7578 | 0.00 |
| *DNTT* | DNA nucleotidylexotransferase | 0.6934 | 0.00 |
| *NR2F2* | nuclear receptor subfamily 2, group F, member 2 | 0.763 | 0.00 |
| *GALNT10* | UDP-N-acetyl-alpha-D-galactosamine:polypeptide N-acetylgalactosaminyltransferase 10 (GalNAc-T10) | 0.7414 | 0.00 |
| *ELMO2* | engulfment and cell motility 2 | 0.7124 | 0.00 |
| *KCTD15* | potassium channel tetramerisation domain containing 15 | 0.7198 | 0.00 |
| *CNPY4* | canopy 4 homolog (zebrafish) | 0.6525 | 0.00 |
| *CADM1* | cell adhesion molecule 1 | 0.751 | 0.00 |
| *KIRREL* | kin of IRRE like (Drosophila) | 0.6114 | 0.00 |
| *CASP3* | caspase 3, apoptosis-related cysteine peptidase | 0.7372 | 0.00 |
| *DPYSL2* | dihydropyrimidinase-like 2 | 0.6179 | 0.00 |
| *CCDC136* | coiled-coil domain containing 136 | 0.6661 | 0.00 |
| *FAM57A* | family with sequence similarity 57, member A | 0.6771 | 0.00 |
| *C15orf48* | chromosome 15 open reading frame 48 | 0.7533 | 0.00 |
| *CASD1* | CAS1 domain containing 1 | 0.6379 | 0.00 |
| *TBC1D8* | TBC1 domain family, member 8 (with GRAM domain) | 0.7611 | 0.00 |
| *IFITM10* | interferon induced transmembrane protein 10 | 0.7412 | 0.00 |
| *TMEM150A* | transmembrane protein 150A | 0.7569 | 0.00 |
| *MLF1* | myeloid leukemia factor 1 | 0.7143 | 0.00 |
| *HEY1* | hairy/enhancer-of-split related with YRPW motif 1 | 0.756 | 0.00 |
| *SLC36A1* | solute carrier family 36 (proton/amino acid symporter), member 1 | 0.6861 | 0.00 |
| *DYNC2H1* | dynein, cytoplasmic 2, heavy chain 1 | 0.697 | 0.00 |
| *TTC38* | tetratricopeptide repeat domain 38 | 0.7246 | 0.00 |
| *LRP12* | low density lipoprotein receptor-related protein 12 | 0.7116 | 0.00 |
| *QKI* | QKI, KH domain containing, RNA binding | 0.6844 | 0.00 |
| *CTNND1* | catenin (cadherin-associated protein), delta 1 | 0.7218 | 0.00 |
| *CNOT6* | CCR4-NOT transcription complex, subunit 6 | 0.6863 | 0.00 |
| *RAB20* | RAB20, member RAS oncogene family | 0.7614 | 0.00 |
| *GNS* | glucosamine (N-acetyl)-6-sulfatase | 0.7626 | 0.00 |
| *SUCLG2* | succinate-CoA ligase, GDP-forming, beta subunit | 0.7436 | 0.00 |
| *PLCG1* | phospholipase C, gamma 1 | 0.7374 | 0.15 |
| *ZFAND2A* | zinc finger, AN1-type domain 2A | 0.7466 | 0.15 |
| *DNAL1* | dynein, axonemal, light chain 1 | 0.7443 | 0.15 |
| *ENOPH1* | enolase-phosphatase 1 | 0.6498 | 0.15 |
| *ZDHHC3* | zinc finger, DHHC-type containing 3 | 0.6249 | 0.15 |
| *CDCA4* | cell division cycle associated 4 | 0.751 | 0.15 |
| *DSCC1* | DNA replication and sister chromatid cohesion 1 | 0.6753 | 0.15 |
| *KLF11* | Kruppel-like factor 11 | 0.7398 | 0.15 |
| *GOLT1B* | golgi transport 1B | 0.6485 | 0.15 |
| *CDKAL1* | CDK5 regulatory subunit associated protein 1-like 1 | 0.64 | 0.15 |
| *MGAT5* | mannosyl (alpha-1,6-)-glycoprotein beta-1,6-N-acetylglucosaminyltransferase | 0.6944 | 0.15 |
| *TMEM51* | transmembrane protein 51 | 0.756 | 0.15 |
| *PSMG3* | proteasome (prosome, macropain) assembly chaperone 3 | 0.7149 | 0.15 |
| *SPOCK1* | sparc/osteonectin, cwcv and kazal-like domains proteoglycan (testican) 1 | 0.7502 | 0.15 |
| *GPR180* | G protein-coupled receptor 180 | 0.6867 | 0.15 |
| *ANXA10* | annexin A10 | 0.7381 | 0.15 |
| *SIRT1* | sirtuin 1 | 0.6819 | 0.15 |
| *PPP2R2C* | protein phosphatase 2, regulatory subunit B, gamma | 0.7294 | 0.15 |
| *THBS2* | thrombospondin 2 | 0.7499 | 0.15 |
| *CXCR4* | chemokine (C-X-C motif) receptor 4 | 0.4942 | 0.15 |
| *LFNG* | LFNG O-fucosylpeptide 3-beta-N-acetylglucosaminyltransferase | 0.7099 | 0.15 |
| *PLXND1* | plexin D1 | 0.7463 | 0.15 |
| *STARD3NL* | STARD3 N-terminal like | 0.6785 | 0.15 |
| *COMMD2* | COMM domain containing 2 | 0.7644 | 0.15 |
| *C6orf48* | chromosome 6 open reading frame 48 | 0.741 | 0.23 |
| *SLC44A2* | solute carrier family 44, member 2 | 0.6748 | 0.23 |
| *MINPP1* | multiple inositol-polyphosphate phosphatase 1 | 0.6606 | 0.23 |
| *SEPN1* | selenoprotein N, 1 | 0.7566 | 0.23 |
| *ZKSCAN3* | zinc finger with KRAB and SCAN domains 3 | 0.7631 | 0.23 |
| *STRA6* | stimulated by retinoic acid 6 | 0.7173 | 0.23 |
| *POLR2A* | polymerase (RNA) II (DNA directed) polypeptide A, 220kDa | 0.7239 | 0.23 |
| *HEATR6* | HEAT repeat containing 6 | 0.7652 | 0.23 |
| *C14orf105* | chromosome 14 open reading frame 105 | 0.6247 | 0.23 |
| *CAMSAP2* | calmodulin regulated spectrin-associated protein family, member 2 | 0.7245 | 0.23 |
| *RALGAPA2* | Ral GTPase activating protein, alpha subunit 2 (catalytic) | 0.7097 | 0.23 |
| *BZW1* | basic leucine zipper and W2 domains 1 | 0.7276 | 0.23 |
| *HMGN3* | high mobility group nucleosomal binding domain 3 | 0.7471 | 0.23 |
| *KLF7* | Kruppel-like factor 7 (ubiquitous) | 0.7158 | 0.23 |
| *PTCH1* | patched 1 | 0.732 | 0.23 |
| *GPRIN2* | G protein regulated inducer of neurite outgrowth 2 | 0.7053 | 0.23 |
| *TTC26* | tetratricopeptide repeat domain 26 | 0.745 | 0.23 |
| *MBOAT1* | membrane bound O-acyltransferase domain containing 1 | 0.6395 | 0.23 |
| *GAL3ST1* | galactose-3-O-sulfotransferase 1 | 0.6833 | 0.23 |
| *SLC6A6* | solute carrier family 6 (neurotransmitter transporter, taurine), member 6 | 0.7133 | 0.23 |
| *MRPS27* | mitochondrial ribosomal protein S27 | 0.619 | 0.23 |
| *PRKACB* | protein kinase, cAMP-dependent, catalytic, beta | 0.7111 | 0.23 |
| *GTF3C4* | general transcription factor IIIC, polypeptide 4, 90kDa | 0.7286 | 0.23 |
| *SNTB2* | syntrophin, beta 2 (dystrophin-associated protein A1, 59kDa, basic component 2) | 0.7551 | 0.23 |
| *CC2D2A* | coiled-coil and C2 domain containing 2A | 0.7685 | 0.23 |
| *AP1M1* | adaptor-related protein complex 1, mu 1 subunit | 0.6272 | 0.23 |
| *YRDC* | yrdC domain containing (E. coli) | 0.7491 | 0.23 |
| *METTL7B* | methyltransferase like 7B | 0.7343 | 0.23 |
| *ATP11A* | ATPase, class VI, type 11A | 0.6435 | 0.23 |
| *SLAMF7* | SLAM family member 7 | 0.6173 | 0.23 |
| *C4orf19* | chromosome 4 open reading frame 19 | 0.7667 | 0.23 |
| *SLC35B4* | solute carrier family 35, member B4 | 0.7108 | 0.23 |
| *TRIM5* | tripartite motif containing 5 | 0.6854 | 0.23 |
| *TOR1B* | torsin family 1, member B (torsin B) | 0.6045 | 0.23 |
| *RFFL* | ring finger and FYVE-like domain containing E3 ubiquitin protein ligase | 0.727 | 0.23 |
| *RGL2* | ral guanine nucleotide dissociation stimulator-like 2 | 0.7639 | 0.23 |
| *VKORC1L1* | vitamin K epoxide reductase complex, subunit 1-like 1 | 0.6333 | 0.23 |
| *GRK5* | G protein-coupled receptor kinase 5 | 0.7553 | 0.23 |
| *PCGF5* | polycomb group ring finger 5 | 0.6603 | 0.23 |
| *NREP* | neuronal regeneration related protein | 0.7191 | 0.23 |
| *USP22* | ubiquitin specific peptidase 22 | 0.7316 | 0.23 |
| *TDRD9* | tudor domain containing 9 | 0.7691 | 0.23 |
| *PLXDC2* | plexin domain containing 2 | 0.7035 | 0.23 |
| *NME6* | NME/NM23 nucleoside diphosphate kinase 6 | 0.7052 | 0.23 |
| *LYN* | v-yes-1 Yamaguchi sarcoma viral related oncogene homolog | 0.7244 | 0.23 |
| *MPP5* | membrane protein, palmitoylated 5 (MAGUK p55 subfamily member 5) | 0.6734 | 0.23 |
| *ARL13B* | ADP-ribosylation factor-like 13B | 0.7387 | 0.23 |
| *RBM22* | RNA binding motif protein 22 | 0.7609 | 0.23 |
| *AK5* | adenylate kinase 5 | 0.7403 | 0.23 |
| *PFKFB3* | 6-phosphofructo-2-kinase/fructose-2,6-biphosphatase 3 | 0.655 | 0.23 |
| *RHBDF2* | rhomboid 5 homolog 2 (Drosophila) | 0.7624 | 0.23 |
| *C5* | complement component 5 | 0.7492 | 0.23 |
| *SLC25A17* | solute carrier family 25 (mitochondrial carrier; peroxisomal membrane protein, 34kDa), member 17 | 0.7273 | 0.23 |
| *CBR4* | carbonyl reductase 4 | 0.6614 | 0.23 |
| *RPRD2* | regulation of nuclear pre-mRNA domain containing 2 | 0.7248 | 0.23 |
| *ETV1* | ets variant 1 | 0.7545 | 0.23 |
| *FBXL20* | F-box and leucine-rich repeat protein 20 | 0.6746 | 0.23 |
| *CLSPN* | claspin | 0.7522 | 0.23 |
| *GABARAPL1* | GABA(A) receptor-associated protein like 1 | 0.6949 | 0.23 |
| *RNF11* | ring finger protein 11 | 0.651 | 0.23 |
| *KIAA1549* | KIAA1549 | 0.7641 | 0.23 |
| *TMEM170A* | transmembrane protein 170A | 0.7034 | 0.23 |
| *NPC2* | Niemann-Pick disease, type C2 | 0.7633 | 0.23 |
| *HMG20A* | high mobility group 20A | 0.7443 | 0.23 |
| *ANGPTL4* | angiopoietin-like 4 | 0.7121 | 0.23 |
| *CA12* | carbonic anhydrase XII | 0.6966 | 0.23 |
| *DDAH1* | dimethylarginine dimethylaminohydrolase 1 | 0.7393 | 0.23 |
| *GLIPR2* | GLI pathogenesis-related 2 | 0.669 | 0.23 |
| *ARPC1A* | actin related protein 2/3 complex, subunit 1A, 41kDa | 0.7153 | 0.23 |
| *ID2* | inhibitor of DNA binding 2, dominant negative helix-loop-helix protein | 0.6389 | 0.23 |
| *CNKSR3* | CNKSR family member 3 | 0.7247 | 0.29 |
| *ACSL3* | acyl-CoA synthetase long-chain family member 3 | 0.6161 | 0.29 |
| *TMEM159* | transmembrane protein 159 | 0.7491 | 0.29 |
| *IGFBP3* | insulin-like growth factor binding protein 3 | 0.51 | 0.29 |
| *KLF12* | Kruppel-like factor 12 | 0.6439 | 0.29 |
| *FAM179B* | family with sequence similarity 179, member B | 0.7624 | 0.29 |
| *GRB14* | growth factor receptor-bound protein 14 | 0.7187 | 0.29 |
| *SPCS3* | signal peptidase complex subunit 3 homolog (S. cerevisiae) | 0.6654 | 0.29 |
| *ZNF460* | zinc finger protein 460 | 0.7122 | 0.29 |
| *ANO6* | anoctamin 6 | 0.6504 | 0.29 |
| *WDYHV1* | WDYHV motif containing 1 | 0.6533 | 0.29 |
| *HMGA2* | high mobility group AT-hook 2 | 0.6533 | 0.29 |
| *RAB3B* | RAB3B, member RAS oncogene family | 0.6579 | 0.29 |
| *KDM4A* | lysine (K)-specific demethylase 4A | 0.7187 | 0.29 |
| *SKP2* | S-phase kinase-associated protein 2, E3 ubiquitin protein ligase | 0.6919 | 0.29 |
| *FRYL* | FRY-like | 0.7269 | 0.29 |
| *TCAIM* | T cell activation inhibitor, mitochondrial | 0.7499 | 0.29 |
| *GPX8* | glutathione peroxidase 8 (putative) | 0.7125 | 0.29 |
| *OR2A4* | olfactory receptor, family 2, subfamily A, member 4 | 0.7548 | 0.29 |
| *ALMS1* | Alstrom syndrome 1 | 0.7251 | 0.29 |
| *SCAMP2* | secretory carrier membrane protein 2 | 0.7642 | 0.29 |
| *GTF3A* | general transcription factor IIIA | 0.7608 | 0.29 |
| *AARS* | alanyl-tRNA synthetase | 0.6698 | 0.29 |
| *CLK4* | CDC-like kinase 4 | 0.7149 | 0.29 |
| *FAM63B* | family with sequence similarity 63, member B | 0.7159 | 0.29 |
| *SFT2D1* | SFT2 domain containing 1 | 0.715 | 0.29 |
| *UGT2B7* | UDP glucuronosyltransferase 2 family, polypeptide B7 | 0.7513 | 0.29 |
| *DAPK1* | death-associated protein kinase 1 | 0.7265 | 0.29 |
| *KLHL36* | kelch-like family member 36 | 0.7578 | 0.29 |
| *NOTCH2* | notch 2 | 0.7324 | 0.29 |
| *KPNA6* | karyopherin alpha 6 (importin alpha 7) | 0.7046 | 0.29 |
| *SPICE1* | spindle and centriole associated protein 1 | 0.7485 | 0.29 |
| *C5orf51* | chromosome 5 open reading frame 51 | 0.7538 | 0.29 |
| *SNCA* | synuclein, alpha (non A4 component of amyloid precursor) | 0.7608 | 0.29 |
| *MFSD6* | major facilitator superfamily domain containing 6 | 0.7414 | 0.29 |
| *TROVE2* | TROVE domain family, member 2 | 0.6876 | 0.29 |
| *FBRS* | fibrosin | 0.7548 | 0.29 |
| *GINS1* | GINS complex subunit 1 (Psf1 homolog) | 0.6836 | 0.29 |
| *GCNT1* | glucosaminyl (N-acetyl) transferase 1, core 2 | 0.7617 | 0.29 |
| *TBX3* | T-box 3 | 0.7425 | 0.29 |
| *CASP2* | caspase 2, apoptosis-related cysteine peptidase | 0.7355 | 0.29 |
| *FAF1* | Fas (TNFRSF6) associated factor 1 | 0.754 | 0.29 |
| *FBXL3* | F-box and leucine-rich repeat protein 3 | 0.7624 | 0.29 |
| *SLC38A7* | solute carrier family 38, member 7 | 0.7362 | 0.29 |
| *PHF5A* | PHD finger protein 5A | 0.7168 | 0.29 |
| *CP* | ceruloplasmin (ferroxidase) | 0.6268 | 0.29 |
| *SLC25A51* | solute carrier family 25, member 51 | 0.6894 | 0.37 |
| *TRIT1* | tRNA isopentenyltransferase 1 | 0.7355 | 0.37 |
| *LRRFIP1* | leucine rich repeat (in FLII) interacting protein 1 | 0.6486 | 0.37 |
| *SPTLC2* | serine palmitoyltransferase, long chain base subunit 2 | 0.766 | 0.37 |
| *CDK6* | cyclin-dependent kinase 6 | 0.6707 | 0.37 |
| *BTN3A2* | butyrophilin, subfamily 3, member A2 | 0.7369 | 0.37 |
| *TEX15* | testis expressed 15 | 0.6149 | 0.37 |
| *ZC3HAV1L* | zinc finger CCCH-type, antiviral 1-like | 0.7677 | 0.37 |
| *ARHGAP29* | Rho GTPase activating protein 29 | 0.7609 | 0.37 |
| *HOXA2* | homeobox A2 | 0.7407 | 0.37 |
| *ARL4C* | ADP-ribosylation factor-like 4C | 0.6674 | 0.37 |
| *GGCX* | gamma-glutamyl carboxylase | 0.7349 | 0.37 |
| *MAPRE3* | microtubule-associated protein, RP/EB family, member 3 | 0.7634 | 0.37 |
| *ST6GAL1* | ST6 beta-galactosamide alpha-2,6-sialyltranferase 1 | 0.7422 | 0.37 |
| *MAPK3* | mitogen-activated protein kinase 3 | 0.7332 | 0.37 |
| *AASDH* | aminoadipate-semialdehyde dehydrogenase | 0.7374 | 0.37 |
| *SHMT1* | serine hydroxymethyltransferase 1 (soluble) | 0.6157 | 0.37 |
| *PLAGL2* | pleiomorphic adenoma gene-like 2 | 0.743 | 0.37 |
| *CDH6* | cadherin 6, type 2, K-cadherin (fetal kidney) | 0.6494 | 0.37 |
| *MME* | membrane metallo-endopeptidase | 0.6929 | 0.37 |
| *SIKE1* | suppressor of IKBKE 1 | 0.7671 | 0.37 |
| *AVEN* | apoptosis, caspase activation inhibitor | 0.7667 | 0.37 |
| *TMCC1* | transmembrane and coiled-coil domain family 1 | 0.7648 | 0.37 |
| *ETV4* | ets variant 4 | 0.6813 | 0.56 |
| *WDR19* | WD repeat domain 19 | 0.7461 | 0.56 |
| *FAM171B* | family with sequence similarity 171, member B | 0.6874 | 0.56 |
| *CAMKK1* | calcium/calmodulin-dependent protein kinase kinase 1, alpha | 0.7458 | 0.56 |
| *HIST1H1D* | histone cluster 1, H1d | 0.7317 | 0.56 |
| *METAP1* | methionyl aminopeptidase 1 | 0.7603 | 0.56 |
| *AGXT2* | alanine--glyoxylate aminotransferase 2 | 0.7579 | 0.56 |
| *HOXA5* | homeobox A5 | 0.6934 | 0.56 |
| *SAMD12* | sterile alpha motif domain containing 12 | 0.7477 | 0.56 |
| *GPR39* | G protein-coupled receptor 39 | 0.7505 | 0.56 |
| *C21orf91* | chromosome 21 open reading frame 91 | 0.7578 | 0.56 |
| *SEMA3C* | sema domain, immunoglobulin domain (Ig), short basic domain, secreted, (semaphorin) 3C | 0.7174 | 0.56 |
| *SHANK2* | SH3 and multiple ankyrin repeat domains 2 | 0.7558 | 0.56 |
| *TLR4* | toll-like receptor 4 | 0.7067 | 0.56 |
| *GUCY1A3* | guanylate cyclase 1, soluble, alpha 3 | 0.6444 | 0.56 |
| *SLC2A3* | solute carrier family 2 (facilitated glucose transporter), member 3 | 0.6991 | 0.56 |
| *CPA4* | carboxypeptidase A4 | 0.5729 | 0.56 |
| *CCL5* | chemokine (C-C motif) ligand 5 | 0.54 | 0.56 |
| *ATP6V0A4* | ATPase, H+ transporting, lysosomal V0 subunit a4 | 0.7554 | 0.74 |
| *GDA* | guanine deaminase | 0.6148 | 0.74 |
| *PEAK1* | NKF3 kinase family member | 0.7182 | 0.74 |
| *TUBA1A* | tubulin, alpha 1a | 0.7078 | 0.74 |
| *MED20* | mediator complex subunit 20 | 0.7343 | 0.74 |
| *G0S2* | G0/G1switch 2 | 0.7575 | 0.74 |
| *ADNP* | activity-dependent neuroprotector homeobox | 0.7484 | 0.74 |
| *ZFHX4* | zinc finger homeobox 4 | 0.7569 | 0.74 |
| *C1orf186* | chromosome 1 open reading frame 186 | 0.7599 | 0.74 |
| *ZNF215* | zinc finger protein 215 | 0.7081 | 0.74 |
| *CHML* | choroideremia-like (Rab escort protein 2) | 0.7404 | 0.74 |
| *NR5A2* | nuclear receptor subfamily 5, group A, member 2 | 0.7233 | 0.74 |
| *GUCY1B3* | guanylate cyclase 1, soluble, beta 3 | 0.5949 | 0.74 |
| *FBXO21* | F-box protein 21 | 0.6945 | 0.74 |
| *ITGA2* | integrin, alpha 2 (CD49B, alpha 2 subunit of VLA-2 receptor) | 0.7509 | 0.74 |
| *BLM* | Bloom syndrome, RecQ helicase-like | 0.6604 | 0.94 |
| *VCAM1* | vascular cell adhesion molecule 1 | 0.6861 | 0.94 |
| *NIPAL1* | NIPA-like domain containing 1 | 0.6934 | 0.94 |
| *PAG1* | phosphoprotein associated with glycosphingolipid microdomains 1 | 0.6965 | 0.94 |
| *NDRG3* | NDRG family member 3 | 0.7679 | 0.94 |
| *NCF2* | neutrophil cytosolic factor 2 | 0.7321 | 0.94 |
| *TNFSF10* | tumor necrosis factor (ligand) superfamily, member 10 | 0.7187 | 0.94 |
| *TFR2* | transferrin receptor 2 | 0.7675 | 0.94 |
| *SLC20A1* | solute carrier family 20 (phosphate transporter), member 1 | 0.7397 | 0.94 |
| *RGS22* | regulator of G-protein signaling 22 | 0.7482 | 0.94 |
| *LOX* | lysyl oxidase | 0.6979 | 0.94 |

**Supplementary Table S3.** Top 10 down-regulated genes in human lung tissues (IPAH vs. Con).

| Gene Symbol | Gene title | Log_2_FC | adj.*P*.Val |
| --- | --- | --- | --- |
| *RNASE2* | Ribonuclease A Family Member 2 | -2.207 | 1.14E-02 |
| *PROK2* | Prokineticin 2 | -2.033 | 3.48E-02 |
| *SERPINF2* | Serpin Family F Member 2 | -1.620 | 1.48E-02 |
| *SPOCK1* | SPARC (Osteonectin), Cwcv And Kazal Like Domains Proteoglycan 1 | -1.609 | 2.01E-02 |
| *TRIB3* | Tribbles Pseudokinase 3 | -1.600 | 1.14E-02 |
| *CD163* | CD163 Molecule | -1.572 | 1.63E-02 |
| *PLA2G3* | Phospholipase A2 Group III | -1.531 | 1.17E-02 |
| *MGST1* | Microsomal Glutathione S-Transferase 1 | -1.520 | 1.14E-02 |
| *KCTD14* | Potassium Channel Tetramerization Domain Containing 14 | -1.494 | 1.48E-02 |
| *ADORA3* | Adenosine A3 Receptor | -1.487 | 1.14E-02 |

**Supplementary Table S4.** Top 10 up-regulated genes in human lung tissues (IPAH vs. Con).

| Gene Symbol | Gene title | Log_2_FC | adj.P.Val |
| --- | --- | --- | --- |
| *CXCL10* | C-X-C Motif Chemokine Ligand 10 | 2.964 | 1.14E-02 |
| *GZMB* | Granzyme B | 2.514 | 1.69E-02 |
| *CCL4L1* | C-C Motif Chemokine Ligand 4 Like 1 | 2.128 | 1.48E-02 |
| *CCL3L3* | C-C Motif Chemokine Ligand 3 Like 3 | 2.108 | 4.59E-02 |
| *APLNR* | Apelin Receptor | 2.087 | 3.42E-02 |
| *DEPP1* | DEPP1 Autophagy Regulator | 2.018 | 2.83E-03 |
| *TNFAIP3* | TNF Alpha Induced Protein 3 | 1.916 | 1.48E-02 |
| *CCL3L1* | C-C Motif Chemokine Ligand 3 Like 1 | 1.796 | 4.35E-02 |
| *IFNG* | Interferon Gamma | 1.788 | 2.33E-02 |
| *PDK4* | Pyruvate Dehydrogenase Kinase 4 | 1.683 | 3.12E-02 |

**Supplementary Table S5.** Physiologic parameter of the human subjects.

| Gene Symbol | Con | IPAH |
| --- | --- | --- |
| Mean age, years | 53.1 | 35.7 |
| Men/women | 4/5 | 2/6 |
| Physiologic parameter, mean ± SD |  |  |
| FVC % | NA | 80.3 ± 15.1 |
| DLCO % | NA | 55.6 ± 22.7 |
| FVC %/DLCO % | NA | 2.02 ± 1.9 |
| Mean PAP, mm Hg | NA | 60.0 ± 13.3 |

Note: NA means not available; FVC means forced vital capacity; DLCO means diffusing capacity for carbon monoxide; PAP means pulmonary artery pressure.
